# Supplementary material for: Guiding protein design choices by per-residue energy breakdown analysis with an interactive web application
Source: Front Mol Biosci. 2023 May 9;10:1178035. doi: 10.3389/fmolb.2023.1178035 (PMC10204868; doi:10.3389/fmolb.2023.1178035)
Supplement: Supplementary file 1 [file DataSheet1.PDF]

Supporting Information for

Guiding Protein Design Choices by Per-Residue Energy Breakdown Analysis with an Interactive Web Application

Felipe Engelberger[1], Jonathan D. Zakary[1], Georg Künze[1,*]
[1] Institute for Drug Discovery, Leipzig University, Brüderstraße 34, 04103 Leipzig, Germany

1. Supporting Code

fast_relax.xml

```
<ROSETTASCRIPTS>
    <SCOREFXNS>
    </SCOREFXNS>
    <TASKOPERATIONS>
    <InitializeFromCommandline name="ifcl" />
    </TASKOPERATIONS>

    <MOVERS>
    <FastRelax name="relax" scorefxn="REF2015" task_operations="ifcl"
repeats="1"/>
    production should be 5 repeats
    FastRelax name="relax" scorefxn="REF2015" task_operations="ifcl" repeats="5"
    </MOVERS>

    <FILTERS>
    </FILTERS>
    <APPLY_TO_POSE>
    </APPLY_TO_POSE>

    <PROTOCOLS>
    <Add mover="relax" />
    </PROTOCOLS>
    <OUTPUT scorefxn="REF2015" />
</ROSETTASCRIPTS>
```

fast_relax.py

```python
def run(
    file_name: str,
    save_score_path: str,
    save_pdb_path: str,
    log_path: str,
    executable: str,
    nstruct: int
) -> None:
    """
    Run the Rosetta Fast Relax Protocol in a Subprocess
    :param file_name:
        The name of the input file
    :param save_score_path:
        The path to save the output score file
    :param save_pdb_path:
        The path to save the output pdb file
    :param log_path:
        The path to save the log file
    :param executable:
        The path to the Rosetta executable
    :return: None
    """
    print(f'Started Job for {file_name} with nstruct: {nstruct}')
    executable = 'rosetta_scripts.static.linuxgccrelease'

    options = [
        f'-s {file_name}',
        f'-native {file_name}',
        f'-parser:protocol lib/XML/fast_relax.xml',
        f'-out:pdb',
        f'-out:path:pdb {save_pdb_path}',
        f'-nstruct {nstruct}',
        f'-out:file:scorefile {save_score_path}'
    ]
    # Add the options Unconstrained and Constrained
    #options.append('-relax:constrain_relax_to_start_coords')

    log = rosetta_simple(executable, options)
```

```
    with open(log_path, 'w') as file:
        file.write(log)
```

energy_breakdown.py

```
def run(
    file_name: str,
    save_path: str,
    log_path: str,
    executable: str,
) -> None:
    """
    Run the Rosetta Energy Breakdown Protocol in a Subprocess
    :param file_name:
        The input PDB file on disk
    :param save_path:
        Location to output the result file
    :param log_path:
        Location to save the log file
    :param executable:
        Filepath of the Rosetta executable to run
    :return: None
    """
    executable = energy_breakdown.static.linuxgccrelease'
    options = [
        f'-in:file:s {file_name}',
        f'-out:file:silent {save_path}'
    ]
    log = rosetta_simple(executable, options)
    with open(log_path, 'w') as file:
        file.write(log)
```

2. Supporting Tables

Table S1: Comparison of the significant energy changes for the structures of designed *Is*PETase mutants and their melting temperatures (T_m) with respect to the wildtype protein.

**Engineered *Is*PETase mutants**	**PDB ID**	**Significant energy change vs WT IsPETase [a]**	**Tm (°C) [d]**	**Reference**
FastPETase	7SH6	-10.2 REU	67.1	[1]
DuraPETase	6KY5	-15.1 REU [b]	77.0	[2]
HotPETase	7QVH	-27.6 REU [c]	82.5	[3]

[a] PDB file of wildtype *Is*PETase was 5XJH
[b] average energy change computed for 2 chains in the PDB file
[c] average energy change computed for 3 chains in the PDB file
[d] Tm value for wildtype *Is*PETase was reported as 46.8°C [4]

3. Supporting References

[1] Lu, H., Diaz, D.J., Czarnecki, N.J. et al. Machine learning-aided engineering of hydrolases for PET depolymerization. Nature 604, 662–667 (2022).

[2] Yinglu Cui, Yanchun Chen, Xinyue Liu, Saijun Dong, Yu'e Tian, Yuxin Qiao, Ruchira Mitra, Jing Han, Chunli Li, Xu Han, Weidong Liu, Quan Chen, Wangqing Wei, Xin Wang, Wenbin Du, Shuangyan Tang, Hua Xiang, Haiyan Liu, Yong Liang, Kendall N. Houk, and Bian Wu ACS Catalysis 2021 11 (3), 1340-1350

[3] Bell, E.L., Smithson, R., Kilbride, S. et al. Directed evolution of an efficient and thermostable PET depolymerase. Nat Catal 5, 673–681 (2022)

[4] Joo, S., Cho, I.J., Seo, H. et al. Structural insight into molecular mechanism of poly(ethylene terephthalate) degradation. Nat Commun 9, 382 (2018).
